# Case Report: Whole exome sequencing reveals a novel frameshift deletion mutation p.G2254fs in COL7A1 associated with autosomal recessive dystrophic epidermolysis bullosa

**DOI:** 10.12688/f1000research.8380.2

**Published:** 2016-07-05

**Authors:** Shamsudheen Karuthedath Vellarikkal, Rijith Jayarajan, Ankit Verma, Sreelata Nair, Rowmika Ravi, Vigneshwar Senthivel, Sridhar Sivasubbu, Vinod Scaria

**Affiliations:** 1 Genomics and Molecular Medicine Unit, CSIR Institute of Genomics and Integrative Biology, Delhi, India; 2 Department of Fetal Medicine, Lifeline Super Specialty Hospital, Kerala, India; 3 GN Ramachandran Knowledge Center for Genome Informatics, CSIR Institute of Genomics and Integrative Biology, Delhi, India; 4 Academy of Scientific and Innovative Research, CSIR-IGIB South Campus, Delhi, India

**Keywords:** Dystrophic epidermolysis bullosa, simplex whole exome sequencing, Collagen VII mutation

## Abstract

Dystrophic epidermolysis bullosa simplex (DEB) is a phenotypically diverse inherited skin fragility disorder. It is majorly manifested by appearance of epidermal bullae upon friction caused either by physical or environmental trauma. The phenotypic manifestations also include appearance of milia, scarring all over the body and nail dystrophy. DEB can be inherited in a recessive or dominant form and the recessive form of DEB (RDEB) is more severe. In the present study, we identify a novel p.G2254fs mutation in *COL7A1* gene causing a sporadic case of RDEB by whole exome sequencing (WES). Apart from adding a novel frameshift Collagen VII mutation to the repertoire of known mutations reported in the disease, to the best of our knowledge, this is the first report of a genetically characterized case of DEB from India.

## Introduction

Dystrophic epidermolysis bullosa (DEB) is an extremely rare subtype of epidermolysis bullosa with an estimated incidence of approximately 6.5 per million newborns. The disease is caused by mutations in collagen VII ( *COL7A1*) ^1^. Collagen VII is a major structural macromolecule of the skin and plays an important component of the anchoring fibrils, which connect the epidermis and dermis of the skin. The disease affects the skin, the mucosa (including that of the oral cavity) and gastrointestinal tract. The blisters are further followed by scarring and development of deformities ^1– 3^.

## Case Report

**Figure 1.**
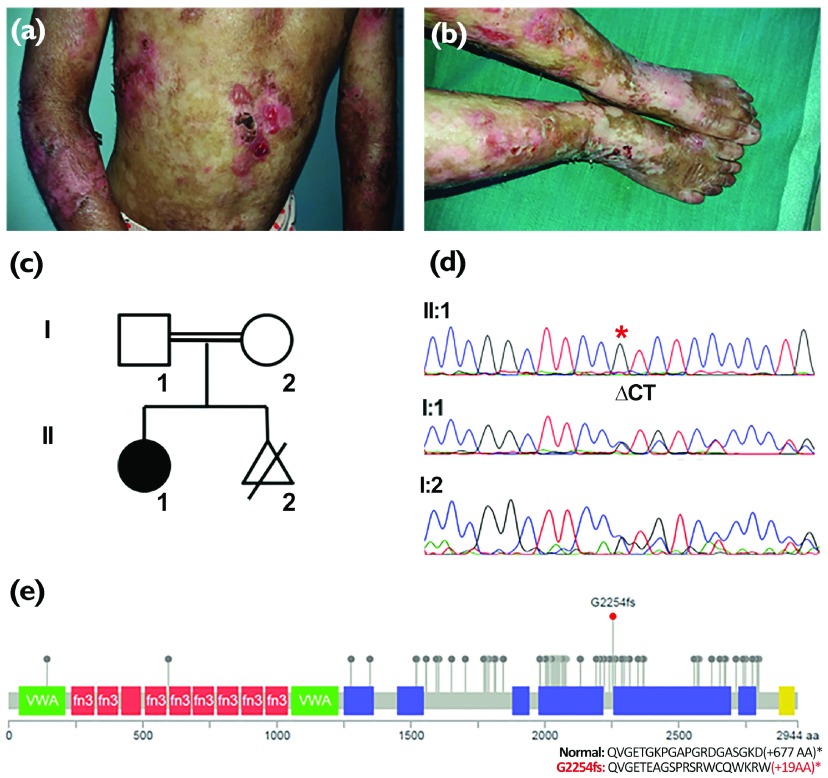
**a**) Hands and thoracic region showing generalized bullae, scarring and milia **b**) Lower legs showing scarring, bullae, milia and characteristic dystrophic nails **c**) Pedigree of the family **d**) The chromatogram depicting capillary sequencing results of c.6759_6760del in the trio. The mutation loci (ΔCT) is highlighted with asterisks **e**) Domain structure of COL7A1 protein showing Von Willebrand factor type A domain (VWA), Fibronectin type III domain (fn3), collagen triple helix domain (blue) and Kunitz domain (yellow). Each needle represents disease causing variation site and the red needle represent p.G2254fs (c.6759_6760del) variation. Panel at the bottom represents COL7A1 p.G2254fs induced PTC compared to the normal protein.

A 4.5-year-old South Indian female child presented to the outpatient clinic with a history of multiple vesicular and bullous lesions induced by trauma since perinatal period. The child was born out of a third degree consanguineous marriage with no known history of similar illness. The child had severe blistering and scarring all over the body, nail dystrophy and milia. The oral mucosa was involved along with tongue blistering, dental calculus, and chipping of teeth with difficulty in opening the mouth. The child also had flexural deformities resulting in contractures and pseudo-syndactyly of the fingers. The clinical picture ( Figure 1a,b) corroborated the diagnosis of dystrophic epidermolysis bullosa (DEB). There is no center in India offering genetic diagnosis for the disease using targeted gene sequencing. Given that targeted gene sequencing can be quite expensive, tedious and time-consuming to standardise, we attempted whole-exome sequencing (WES). Moreover no background genetic map of mutations in the disease from India was available. Previous reports, including from our laboratory suggest WES as an alternative to traditional approaches; WES is fast, less tedious, and cost-effective and also provides a holistic view of the mutation spectrum in the patient ^4– 6^.

Approximately 5 ml of blood was collected from the affected individual and the parents after obtaining signed informed consent and approval from the institutional ethical committee (BSC0212 IHECC proposal No.08). Genomic DNA was isolated by using salting out method ^7^. 50ng of high quality DNA was used for whole exome sample preparation using a Nextera (Illumina Inc, USA) expanded exome kit according to manufacturer supplied instruction. The exome was sequenced using Illumina Hiseq2500 according to the manufacturer’s protocols (Illumina Inc, USA). Paired-end reads of 150 bases were generated, which was quality and adapter trimmed at a Phred quality score of 20. Alignment was performed on the human reference genome (hg19) using Burrows-Wheeler Alignment (version 0.5.10-evan.9) ^8^. The mean mapped coverage on target region was 12.2x. Variants were called using Platypus pipelines (version 0.7.9.1) ^9^. Analysis revealed a novel homozygous frameshift deletion (chr3:g.48610366CT>-) c.6759_6760del (p.G2254fs) in *COL7A1* gene. The c.6759_6760del was predicted to be deleterious (confidence score 0.858) and introduce a premature termination codon (PTC) at 2273 ^th^ amino acid position according to SIFT ^10^. Homozygous PTCs in *COL7A1* is previously reported to reduce overall stability of anchoring filaments and cause mild to very severe generalised RDEB ^1^. Secondary structure analysis shows that p.G2254fs resultant PTC leads to loss of function of several collagen triple helix repeats and kunitz domain ( Figure 1e). We also found a homozygous nonsynonymous variation c.5716C>T (p.P1906S) in COL7A1, which was predicted to be ‘tolerated’ by SIFT (0.5) ^11^.

The variant was verified independently using capillary sequencing in the child and parents. The variant was not found in ExAC or our internal cohort of 122 exomes, confirming its rarity and novelty. Parents were provided detailed genetic counselling by the consulting clinical geneticist.

## Discussion

Dystrophic EB could be inherited in both recessive and dominant form ^1^. Several cases of DEB have been reported from India. A recent paper reported a cohort of 17 DEB patients using immunofluorescence mapping ^12^, though the patients were not genetically characterized. Our earlier report characterized a novel mutation in *KRT5* associated with epidermolysis bullosa (EB) simplex in West India ^6^. Taken together, we suggest a large and potentially uncharacterized repertoire of genetic variations causing EB in India, which might benefit from genetic screening approaches.

In this study, we show the application of next-generation sequencing to identify the mutation in a sporadic case of autosomal recessive EB in clinical settings. Apart from adding a novel frameshift collagen VII deletion mutation to the repertoire of known mutations in the disease, to the best of our knowledge, this is the first report of a genetically characterized patient of DEB from India. We suggest that next-generation sequencing approach would significantly benefit the understanding and genetic characterization of this rare disease in India.

## Consent

Written informed consent was obtained from the parent of the patients for publication of this case report and any accompanying images and/or other details that could potentially reveal the patient’s identity.

## Data availability

The data referenced by this article are under copyright with the following copyright statement: Copyright: © 2016 Karuthedath Vellarikkal S et al.

The raw exome sequencing data are available at the NCBI Sequence Read Archive ( http://www.ncbi.nlm.nih.gov/sra), accession number SRX1584466.
