# Supplementary material for: Case Report: Whole exome sequencing reveals a novel frameshift deletion mutation p.G2254fs in COL7A1 associated with autosomal recessive dystrophic epidermolysis bullosa
Source: F1000Res. 2016 Jul 5;5:900. Originally published 2016 May 17. [Version 2] doi: 10.12688/f1000research.8380.2 (PMC4926754; doi:10.12688/f1000research.8380.2)
Supplement: Peer review history

# Peer review: Referee response for version 2

**DOI:** 10.5256/f1000research.9847.r14976

**Published:** 2016-07-13

**Authors:** Robert Sidbury

**Affiliations:** 1 Dermatology Division, Seattle Children, Seattle, WA, USA

The authors have responded satisfactorily.

I have read this submission. I believe that I have an appropriate level of expertise to confirm that it is of an acceptable scientific standard.

# Peer review: Referee response for version 1

**DOI:** 10.5256/f1000research.9013.r14177

**Published:** 2016-06-27

**Authors:** Mohamed Badawy Abdel-Naser

**Affiliations:** 1 Klinik für Dermatologie, Venerologie und Allergologie Städtisches Klinikum Dessau, Dessau, Germany

- Apart from few typo errors, the manuscript is well written and informative.
- There are few too strong statements, e.g.,  "The disease also predisposes individuals to development of skin cancer and it is estimated that almost all affected members develop cancers in the third or fourth decade of life". Perhaps authors can insert a reference (s) that supports this statement.
- Fig. 1. needs some corrections. Please insert (a) to refer to the upper limb, thorax and abdomen (not hands and thoracic region). b. should refer to legs and feet.
- Is the mentioned "our internal cohort of 122 exomes" published or available online? Furthermore, they may mention some of the weaknesses of the Nextera platform, such as coverage bias.
- In the discussion sections, authors may mention the clinical relevance of their finding.

I have read this submission. I believe that I have an appropriate level of expertise to confirm that it is of an acceptable scientific standard.

# Peer review: Referee response for version 1

**DOI:** 10.5256/f1000research.9013.r14364

**Published:** 2016-06-14

**Authors:** Regina Fölster-Holst

**Affiliations:** 1 Dermatologische Klinik, Universität Kiel, Kiel, Germany

The authors describe a 5 year old boy with dystrophic epidermolysis bullosa (DEB), which is due to a novel mutation in the *COL7A1 *gene. The case is the first report of a genetically characterized case of DEB from India.

 It is a well written manuscript which is worthy to be indexed with *F1000Research*. To understand the new mutation as the cause of severe DEB in the boy, it would be interesting to know what are the differences to other mutations of the *COL7A1 *gene. In other words, what does this mean on the protein level?

I have read this submission. I believe that I have an appropriate level of expertise to confirm that it is of an acceptable scientific standard.

# Peer review: Referee response for version 1

**DOI:** 10.5256/f1000research.9013.r14175

**Published:** 2016-06-06

**Authors:** Robert Sidbury

**Affiliations:** 1 Dermatology Division, Seattle Children, Seattle, WA, USA

Though the authors distinguish dominant (DDEB) and recessive (RDEB) subtypes of dystrophic epidermolysis bullosa (DEB) at the outset in their abstract, they lapse into speaking of DEB in more monolithic terms later and conflating findings that are specific in some cases only to one  subtype.  There is a reason for this as there is considerable overlap but there are differences particularly with certain features like the propensity for developing skin cancer.  The authors state  "the development of  skin cancer....in almost all affected members in the third or fourth decade of life."  This is true for RDEB phenotype but not DDEB in whom the development of squamous cell carcinoma as well as the distinctive psuedosyndactyly type of scarring much less common.  This may not be the forum for parsing such details but this struck me.

 Similarly, while I realize this is not an EB review article a brief internal reference to the Vander Oever article might allow interested readers easy access to a therapeutic update.

 Finally,  I would want someone other than I with expertise in the genetic methods used to weigh in on their suitability. The methods and results to my untrained --relative to a geneticist--- eye appear sound.

 Otherwise I approve indexing this article.

I have read this submission. I believe that I have an appropriate level of expertise to confirm that it is of an acceptable scientific standard, however I have significant reservations, as outlined above.

## References

[1] McGrathJA: Recently Identified Forms of Epidermolysis Bullosa. *Ann Dermatol*.2015;27(6) : 10.5021/ad.2015.27.6.658 658-66 10.5021/ad.2015.27.6.658 26719633PMC4695416

[2] Vanden OeverMJTolarJ: Advances in understanding and treating dystrophic epidermolysis bullosa. *F1000Prime Rep*.2014;6: 10.12703/P6-35 35 10.12703/P6-35 24860657PMC4017907

# Response

**Published:** 2016-06-27

**Authors:** Vinod Scaria

**Affiliations:** Institute of Genomics and Integrative Biology, Delhi, India

Dear Robert Sidbury,

 Thank you for reviewing our article and your valuable comments. We have incorporated your suggestions in the recent version.

 Thanks and Regards

 Authors
